# Protein kinase CK2 inhibition suppresses neointima formation via a proline-rich homeodomain-dependent mechanism

**DOI:** 10.1016/j.vph.2017.09.004

**Published:** 2017-12

**Authors:** K.S. Wadey, B.A. Brown, G.B. Sala-Newby, P.-S. Jayaraman, K. Gaston, S.J. George

**Affiliations:** a School of Clinical Sciences, University of Bristol, Research Floor Level 7, Bristol Royal Infirmary, Bristol BS2 8HW, UK; b Department of Biochemistry, University of Bristol, Bristol BS8 1TD, UK; c Division of Immunity and Infection, College of Medicine, University Birmingham, Edgbaston, Birmingham B15 2TT, UK

**Keywords:** BCA, Bicinchoninic Acid, bFGF, basic fibroblast growth factor, DMEM, Dulbecco's modified essential medium, FCS, foetal calf serum, Hex/Hhex, haematopoietically expressed homeobox, HUVECs, human umbilical ven endothelial cells, HSaVECs, human saphenous vein endothelial cells, K66, 1-carboxymethyl-2-dimethylamino-4,5,6,7-tetrabromo-benzimidazole, PDGF-BB, platelet-derived growth factor-BB, pfu, plaque forming units, PRH, proline-rich homeodomain, SDS, sodium dodecyl sulphate, siRNA, silencing RNA, TBB, 4,5,6,7-tetrabromobenzotriazole, VSMC, vascular smooth muscle cell, Vascular smooth muscle, Vein graft, In-stent restenosis, Atherosclerosis, PRH, CK2

## Abstract

Neointimal hyperplasia is a product of VSMC replication and consequent accumulation within the blood vessel wall. In this study, we determined whether inhibition of protein kinase CK2 and the resultant stabilisation of proline-rich homeodomain (PRH) could suppress VSMC proliferation. Both silencing and pharmacological inhibition of CK2 with K66 antagonised replication of isolated VSMCs. SiRNA-induced knockdown as well as ectopic overexpression of proline-rich homeodomain indicated that PRH disrupts cell cycle progression. Mutation of CK2 phosphorylation sites Ser^163^ and Ser^177^ within the PRH homeodomain enabled prolonged cell cycle arrest by PRH. Concomitant knockdown of PRH and inhibition of CK2 with K66 indicated that the anti-proliferative action of K66 required the presence of PRH. Both K66 and adenovirus-mediated gene transfer of S163C:S177C PRH impaired neointima formation in human saphenous vein organ cultures. Importantly, neither intervention had notable effects on cell cycle progression, cell survival or migration in cultured endothelial cells.

## Graphical abstract

**Figure:**
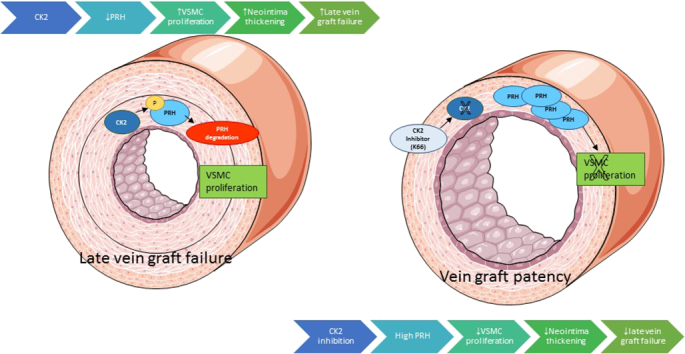

## 1 Introduction

Vessel occlusion following neointima development restricts the therapeutic potential of peripheral and coronary artery bypass grafts, and percutaneous coronary interventions. Accumulation of vascular smooth muscle cells (VSMCs) within the neointimal compartment greatly facilitates the formation of restenotic lesions via both contributing to its occlusive mass and serving as a foundation for superimposed, accelerated atherosclerosis [1], [2]. Intervening with modulators of VSMC proliferation is hence considered a pragmatic approach in designing preventative therapies against vein graft degeneration and in-stent restenosis.

Protein kinase CK2 is a ubiquitously expressed and highly pleiotropic enzyme implicated in a diverse range of cellular functions including such indices as cell cycle progression, viability and motility [3]. Aberrant activity has predictably been associated with malignant transformation and aggressive tumour growth, with T-cell lymphomas [4], non-small cell lung cancer [5], prostate cancer [6], and tumors of the head, neck, mammary gland and kidney [7], [8], [9] being documented examples. Emodin, DRB, DDZ and TBB, both naturally derived and synthetic inhibitors of CK2, have been reported to induce cell cycle arrest in isolated VSMCs [10], [11], [12], though in consideration of the promiscuity of these compounds this action could not be solely ascribed to suppression of CK2 activity.

The proline-rich homeodomain (PRH), also referred to as haematopoietically expressed homeobox (Hex/Hhex), is a multifunctional transcription factor critical for embryonic and tissue development - notably of the haematopoietic and vascular lineages [13], [14], [15]. PRH can operate as either a potent facilitator or inhibitor of cellular proliferation dependent on context; though in adult epithelial and vascular tissues, is most often reported to mediate cell cycle arrest and exhibit tumour suppressor properties [14], [16], [17], [18]. Our previous studies have reported that protein kinase CK2-dependent phosphorylation of PRH at residues Ser^163^ and Ser^177^ abrogates DNA-binding potential and transcriptional regulation activity, reduces nuclear retention and lowers stability via directing PRH for proteasome-mediated proteolysis [19], [20]. By this action, CK2 alleviates PRH-mediated suppression of cell cycle progression in human leukaemic myeloid K562 cells [19]. We hypothesize therefore that inhibition of CK2 leads to suppression of VSMC proliferation via PRH. This hypothesis contradicts what was postulated by Sekiguchi and colleagues, where it was proposed that PRH facilitates neointima formation by participating in the de-differentiation of medial VSMC [21].

## 2 Materials and methods

All reagents were purchased from Sigma Aldrich Corporation unless otherwise stated.

### 2.1 Isolation and culture of primary rat aortic VSMCs

The housing and care of all animals was undertaken in accordance with the guidelines and regulations of the University of Bristol and the United Kingdom Home Office. This study conforms to the *Guide for the Care and Use of Laboratory Animals* published by the US National Institutes of Health (NIH Publication No. 85-23, revised 1996). VSMCs were grown from thoracic and abdominal rat aorta employing the explant method described previously [22]. VSMCs were cultured in Dulbecco's modified essential medium (DMEM; GE Healthcare) supplemented with 100 μg/ml penicillin and 100 IU/ml streptomycin, 2 mM l-glutamine, 8 μg/ml gentamicin and 10% (v/v) foetal calf serum (FCS) (PAA laboratories); cells between passages 2 and 9 were utilised for experiments. Recombinant human platelet-derived growth factor-BB (PDGF-BB), recombinant human basic fibroblast growth factor (bFGF), and recombinant mouse Wnt-4 were purchased from R & D Systems. Pharmacological inhibitors of protein kinase CK2, TBB (4,5,6,7-tetrabromobenzotriazole) and K66 (1-carboxymethyl-2-dimethylamino-4,5,6,7-tetrabromo-benzimidazole), were acquired from Sigma Aldrich and Merck Millipore, respectively.

### 2.2 Culture of HUVECs and HSaVECs

Human umbilical vein endothelial cells (HUVECs) and human saphenous vein endothelial cells (HSaVECs) were obtained from Promocell. Cells were cultured in endothelial cell growth medium (Promocell; C-22010) or endothelial cell basal medium (Promocell; C-22210) supplemented with 100 μg/ml penicillin and 100 IU/ml streptomycin; basal medium was additionally supplemented with 0.5–2.0% FCS. Additional components of the complete endothelial cell growth medium included 20 μl/ml foetal calf serum, 4 μl/ml endothelial cell growth supplement, 0.1 ng/ml recombinant human epidermal growth factor, 1 ng/ml recombinant human bFGF, 90 μg/ml heparin and 1 μg/ml hydrocortisone.

### 2.3 Preparation and culture of human saphenous vein organ cultures and VSMCs

To induce intima formation human saphenous vein segments were subjected to organ culture as described previously [23]. Surplus segments of surgically prepared human saphenous vein obtained from consenting patients (Ethics number REC: 11/SW/0154) were collected and dissected in 25 mM Hepes-buffered RPMI 1640 culture medium (Gibco) supplemented with 100 μg/ml penicillin and 100 IU/ml streptomycin, 2 mM l-glutamine, 8 μg/ml gentamicin and 10% (v/v) FCS. Adventitia was carefully removed to minimise fibroblast growth, vein opened along its longitudinal axis and transverse segments cut to yield 5–10 mm segments. Vein segments were pinned down onto mesh in Sylgard resin-coated petri dishes and cultured in sodium bicarbonate-buffered RPMI 1640 (Gibco) supplemented with 100 μg/ml penicillin and 100 IU/ml streptomycin, 2 mM l-glutamine, 8 μg/ml gentamicin, 30% (v/v) FCS and 10 μM BrdU. Culture medium was replenished every 2–3 days. On day 14, venous material was either fixed in 10% (v/v) formalin/PBS for 24 h in preparation for embedding in paraffin-wax, or subjected to protein extraction. The latter was achieved by chopping vein segments in 100 μl of sodium dodecyl sulphate (SDS) lysis buffer (50 mM Tris-HCl (pH 8), 10% (v/v) glycerol, 5% (w/v) SDS) and keeping on ice for 30 min prior to micro-centrifuging at 14,000 *g* for 5 min to remove debris. VSMCs were cultured from segments of saphenous vein as previously described [24].

### 2.4 Adenovirus-mediated gene transfer

Adenoviral constructs expressing β-galactosidase, c-myc-tagged wild-type PRH and S163C:S177C PRH were prepared using the shuttle vector pDC 515 (Microbix Biosystems) and made replication-deficient by site-specific FLP-mediated recombination. Viral preparations were purified on a caesium chloride gradient and the number of plaque forming units (pfu) per ml calculated by end point dilution in HEK293 cells. Recombinant adenoviruses were diluted in culture medium to a final concentration of 1 × 10^8^ pfu/ml; regarding cultured cells, culture medium was refreshed 18 h later. Gene transfer was validated by both RT-qPCR and Western blotting.

### 2.5 Amaxa nucleofection for gene transfer and silencing

Expression vectors and small interfering RNAs (siRNAs) were introduced into rat aortic VSMCs utilising a Nucleofector device and the Amaxa Basic Nucleofector Kit for primary mammalian smooth muscle cells (Lonza; VPI-1004) in accordance with manufacturer's instructions. For overexpression studies, 1 × 10^6^ cells were subjected to nucleofection with 5 μg of eGFP-encoding plasmids (control), or 2.5 μg of eGFP- and 2.5 μg of wild-type/S163C:S177C PRH-expressing plasmids using the Nucleofector D-033 programme. Similarly, gene silencing was achieved through delivery of 120–480 pmol Allstars negative control siRNAs (Qiagen; 1027281), rat/human PRH (Qiagen; SI00042056), rat CK2α (Qiagen; SI02007180, SI02007159) or rat CK2α′ (Qiagen; SI04730628, SI04730621) siRNAs. Significant depletion of PRH protein required a double knockdown, where VSMCs were Amaxa nucleofected with 240 ng Allstars negative control siRNAs or 240 ng PRH-specific siRNAs, incubated for 72 h in 10% FCS culture medium, before being subjected to nucleofection again with identical conditions. Gene transfer and silencing were validated by both RT-qPCR and Western blotting.

### 2.6 Gene transfer with jet-PEI-HUVEC transfection reagent

HUVECs were seeded in fibronectin (50 μg/ml)-coated wells and incubated overnight in endothelial cell growth medium. Culture medium was replaced with 2% (v/v) endothelial cell basal medium. Plasmids/jetPEI-HUVEC complexes were formulated in accordance with manufacturer's instruction such that the N/P ratio equated to 5; HUVECs were incubated with 100 μl of the prepared plasmid/jetPEI-HUVEC complexes for 4 h, then culture medium replaced with endothelial cell growth medium. Gene transfer was validated by both RT-qPCR and Western blotting.

### 2.7 Scratch wound assay

Cells grown in wells to confluency were scratched using a 1 ml pipette tip to draw an X-shaped wound, then culture medium replaced and supplemented with 2 mM hydroxyurea to inhibit proliferation as described previously [24]. Wound healing was allowed for 24 h and the migrated distance measured at 20 points along the wound edge using Q-Capture Pro Imaging software.

### 2.8 Proliferation assays – BrdU incorporation and Click-iT EdU Imaging

Culture medium was supplemented with 10 μM BrdU or EdU and incorporation quantified via immunocytochemistry/immunofluorescence for BrdU, or application of the Click-iT EdU Alexa Fluor 488 Imaging Kit (Molecular Probes; C10337), in accordance with manufacturer's instructions.

### 2.9 Immunocytochemistry

Cells seeded on glass coverslips were fixed in 3% (w/v) paraformaldehyde/PBS for 10 min and permeabilised with 0.1–1.0% (v/v) Triton X-100/PBS. Immunocytochemistry for BrdU required DNA denaturation which was achieved by incubation with 2N HCl for 30 min at 37 °C. Cells were then blocked with 20% (v/v) goat serum/PBS for 30 min, and probed with primary antibody diluted in 1% (w/v) BSA/PBS overnight at 4 °C or for 1 h at 37 °C. Primary antibodies included: 8.6 μg/ml anti-BrdU IgG (Sigma; B2531) and 1 μg/ml anti-cleaved caspase-3 IgG (R&D Systems; MAB835). Subsequently, cells were incubated with appropriate biotinylated secondary antibody diluted 1:200 in 1% (w/v) BSA/PBS for 45 min, then Dylight 488/594 Streptavidin (Vector Laboratories; SA-5488-1/SA-5594-1) or Extravidin-HRP diluted 1:200 in 1% (w/v) BSA/PBS for 45 min. Prolong Gold Antifade Reagent with DAPI (Molecular Probes; P36931) was utilised for mounting, or cells were incubated with Fast DAB Peroxidase Substrate, stained with haematoxylin and mounted in VectaMount Permanent Mount Medium (Vector Laboratories; H-5000).

### 2.10 Immunohistochemistry/immunofluorescence

Citric acid antigen retrieval was performed on deparaffinised and rehydrated tissue. For BrdU detection DNA denaturation was achieved by incubation with 125 U/ml benzonase, 1 mM Mg^2 +^ in PBS for 2 h at 37 °C. Sections were blocked in either 20% (v/v) goat serum/PBS or Image-iT FX Signal Enhancer (Molecular Probes; I36933), then probed with primary antibodies diluted in 1% (w/v) BSA/PBS overnight at 4 °C. Primary antibodies included: 88 ng/ml anti-α-smooth muscle cell actin IgG (Dako; M0851), 8.6 μg/ml anti-BrdU IgG (Sigma; B2531), 4 μg/ml anti-cleaved caspase-3 IgG (R&D Systems; MAB835), 22.4 ng/ml anti-c-myc-tag IgG (Cell Signalling; 2276), 8.5 μg/ml anti-phospho-CK2 substrate (motif pS/pTDXE) IgG (Cell Signalling; 8738), 2.5 μg/ml anti-PRH IgG (Abcam; ab34222) and 1:1000 anti-phospho-PRH IgG (in-house). Non-immune IgG was used as negative control. Subsequently, sections were incubated with appropriate biotinylated secondary antibody diluted 1:200 in 1% (w/v) BSA/PBS for 45 min, then Dylight 488/594 Streptavidin (Vector Laboratories; SA-5488-1/SA-5594-1) or Extravidin-HRP diluted 1:200 in 1% (w/v) BSA/PBS for 45 min. Prolong Gold Antifade Reagent with DAPI (Molecular Probes; P36931) was utilised for mounting, or sections were incubated with Fast DAB Peroxidase Substrate, stained with haematoxylin, dehydrated and mounted in VectaMount Permanent Mount Medium (Vector Laboratories; H-5000). Staining was quantified by calculating either the percentage of positive cells or via pixel analysis using Image-Pro Image software.

### 2.11 Histological processing

Formalin-fixed human saphenous vein organ cultures were transferred to PBS, then subsequently process and embedded in paraffin wax using the Shandon Excelsior Tissue Processor (Thermo Electron Corporation). Section of 3 μm thickness were cut on a microtome and dried onto Superfrost Plus Microscope Slides (Thermo Scientific; J1800AMNZ).

### 2.12 Western blotting

Cells were lysed in SDS lysis buffer (50 mM Tris-HCl (pH 8), 10% (v/v) glycerol, 5% (w/v) SDS) and protein concentrations calculated using the Micro Bicinchoninic Acid (BCA) Assay Kit (Thermo Scientific; 23235) in accordance with manufacturer's instructions. Western blotting was performed using the Bio-Rad Mini Format 1-D Electrophoresis System. One volume Laemmli Sample Buffer (Bio-Rad; 161-0737) with 5% (v/v) β-mercaptoethanol was added to 5-10 μl of lysate, for which protein concentration was normalised with HPLC water. Samples and BLUeye Prestained Protein Ladder (Geneflow; S6-0024) were heated at 95 °C for 5 min before loading onto Mini-PROTEAN TGX Gels (Bio-Rad; 456-1084) or Mini-PROTEAN TGX Stain-Free Gels (Bio-Rad; 456-8084), and being electrophoresed in 1 × Tris/Glycine/SDS running buffer (Bio-Rad; 161-0772) at 300 V for 20 min. Protein was normalised against either β-actin or in-gel stain-free bands visualised via a 5 min exposure to UV light. Protein was then transferred to Trans‑Blot Mini/Midi Nitrocellulose Membranes (Bio-Rad; 170-4158/170-4150) using the Trans-Blot Turbo Transfer Starter System (Bio-Rad; 170-4155).

Nitrocellulose membranes were blocked in 5% fat-free milk powder/TBST (20 mM Tris, 137 mM NaCl, 0.1% (v/v) Tween 20; pH 7.6), incubated overnight at 4 °C with diluted primary antibody, washed with TBST, probed with appropriate HRP-conjugated secondary antibody, and washed with TBST before signal detection with Luminata Forte Western HRP substrate (Merck Millipore; WBLUF0100). Primary antibodies included: 0.11 μg/ml anti-β-actin IgG (Sigma; A5316), 20 ng/ml anti-CK2α IgG (Cell Signalling; 2656), 1:500 anti-CK2α’ IgG (Abcam; ab135245), 0.85 μg/ml anti-phospho-CK2 substrate (motif pS/pTDXE) IgG (Cell Signalling; 8738), 0.25 μg/ml anti-PRH IgG (Abcam; ab34222) and 1:2000 anti-phospho-PRH IgG (in-house).

### 2.13 Statistics

Data were analysed by Student *t*-tests for two groups, or ANOVA and Student-Newman-Keuls Multiple Comparisons post hoc test was utilised for more than two group analyses. The experiments were repeated 3–7 times with different batches of cells or samples of tissue, and findings were considered statistically significant when p < 0.05.

## 3 Results

### 3.1 Pharmacological inhibition of protein kinase CK2 retarded VSMC proliferation in vitro

**Fig. 1:**
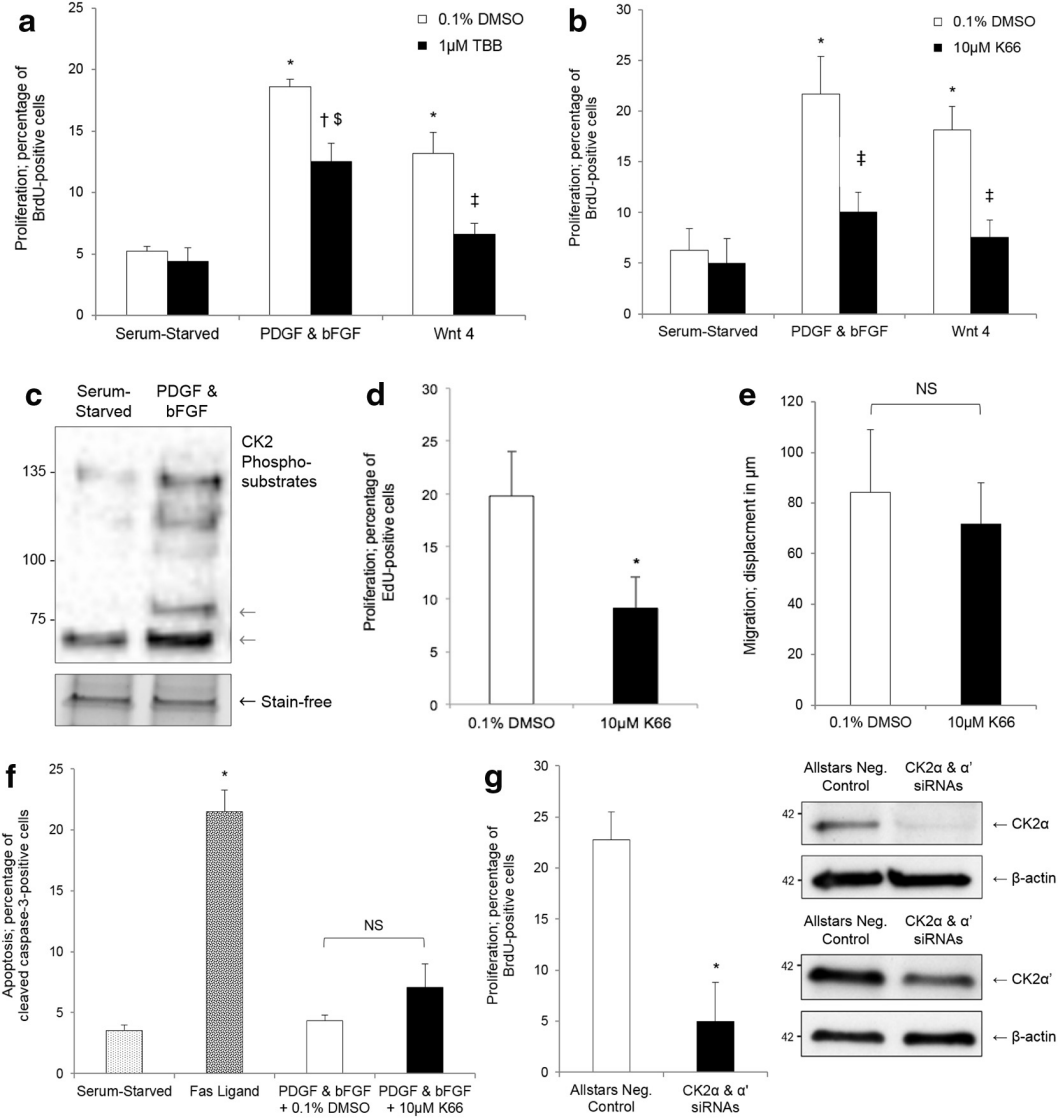
Inhibition and silencing of protein kinase CK2 reduced proliferation of cultured VSMCs. Quiesced rat aortic VSMCs were stimulated with either 20 ng/ml PDGF-BB and 20 ng/ml bFGF, or 500 ng/ml Wnt-4, or remained serum-starved, in the presence of a CK2 inhibitor (a, 1 μM TBB; b, 10 μM K66) or vehicle control (0.1% DMSO). Proliferation was assessed by quantification of BrdU incorporation after 24 h. * indicates significant difference compared to serum-starved – 0.1% DMSO, n = 3. † indicates significant difference compared to PDGF & bFGF – 0.1% DMSO, n = 3. ‡ indicates significant difference compared to Wnt-4 – 0.1% DMSO, n = 3. $ indicates significant difference compared to serum-starved – 0.1% DMSO and serum-starved - 1 μM TBB/10 μM K66, n = 3, p < 0.05, ANOVA and Student Newman Keuls post hoc test. c, Quiesced human saphenous vein VSMCs were stimulated with 20 ng/ml PDGF-BB and 20 ng/ml bFGF, or serum starved, for 24 h. Representative Western blot for CK2 substrates with a consensus pS/pTDXE motif, n = 3. Grey arrows indicate quantified bands. d, Quiesced human saphenous vein VSMCs were stimulated with 20 ng/ml PDGF-BB and 20 ng/ml bFGF in the presence of 10 μM K66 or 0.1% DMSO, and proliferation assessed by quantification of EdU incorporation after 24 h. * indicates significant difference compared to 0.1% DMSO, n = 5, p < 0.05, Students *t*-test. e, Quantification of migration using the scratch wound assay in VSMCs cultured in the presence of CK2 inhibitor (10 μM K66) or vehicle control (0.1% DMSO), n = 4. f, Rat aortic VSMCs were stimulated with 20 ng/ml PDGF-BB and 20 ng/ml bFGF in the presence of 10 μM K66 or 0.1% DMSO for 24 h and then immunocytochemistry for cleaved caspase-3 performed to quantify apoptosis. 200 ng/ml Fas ligand was used a positive control. * indicates significant difference compared to serum-starved control, n = 5, p < 0.05, ANOVA and Student Newman Keuls post hoc test. NS denotes not significant. g, Quiesced rat aortic VSMCs were subjected to nucleofection with either Allstars negative control, or CK2α & α’ siRNAs, then stimulated with both 20 ng/ml PDGF-BB and 20 ng/ml bFGF. Proliferation was assessed via quantification of BrdU incorporation between 48 and 72 h post-introduction of siRNAs. * indicates significant difference compared to Allstars Negative Control, n = 3, p < 0.05, Students *t*-test. Western blots show validation of silencing of CK2α & α′ catalytic subunits, representative of n = 3.

Primary cultures of rat aortic VSMCs were serum-starved for 72 h, then quiescent cells stimulated with 20 ng/ml recombinant human PDGF-BB and 20 ng/ml recombinant human bFGF, or 500 ng/ml recombinant mouse Wnt-4, or serum-starved for a further 24 h. Concurrently, cells were treated with 0.1% DMSO vehicle control or synthetic CK2 inhibitors - 1 μM TBB (4,5,6,7-tetrabromobenzotriazole) or 10 μM K66 (1-carboxymethyl-2-dimethylamino-4,5,6,7-tetrabromo-benzimidazole) in 0.1% DMSO. Both compounds have low promiscuity and high efficacy at the applied concentrations [25]. As assessed by quantifying BrdU incorporation, inhibition of CK2 with K66 significantly suppressed VSMC proliferation, with the rate of cell replication reduced to basal rates while TBB only did this with Wnt4 stimulated cells (Fig. 1A and B). K66 was a more potent anti-proliferative agent than TBB (PDGF-BB & bFGF; 53.8 ± 0.8% inhibition vs. 33.2 ± 7.1%, p < 0.05). To validate activation of CK2 in response to a growth stimulus, quiescent human saphenous vein VSMCs were treated with 20 ng/ml recombinant human PDGF-BB and 20 ng/ml recombinant human bFGF, or serum-starved, for 24 h; Western blotting was subsequently performed for proteins containing a pS/pTDXE motif, a CK2 phosphorylation consensus sequence. Phosphorylation of CK2 substrates was significantly elevated in response to PDGF-BB and bFGF stimulation (2.6 ± 0.3-fold induction, p < 0.05), demonstrating activation of protein kinase CK2 (Fig. 1C). K66 also impaired cell cycle progression in isolated human saphenous vein VSMCs: quiescent cells were stimulated with 20 ng/ml recombinant human PDGF-BB and 20 ng/ml recombinant human bFGF in the presence of 10 μM K66 or 0.1% DMSO, and cell replication monitored by quantification of EdU incorporation for 24 h (Fig. 1D). These data suggest that the action of K66 is not species-specific. Scratch wounds assays and immunocytochemistry for cleaved caspase-3 indicated that K66 did not influence cell migration (Fig. 1E) or viability (Fig. 1F) in rat aortic VSMCs. Additionally, siRNA-mediated silencing of CK2 α and α’ catalytic subunits suppressed VSMC proliferation (Fig. 1G).

### 3.2 PRH antagonised VSMC proliferation in vitro

**Fig. 2:**
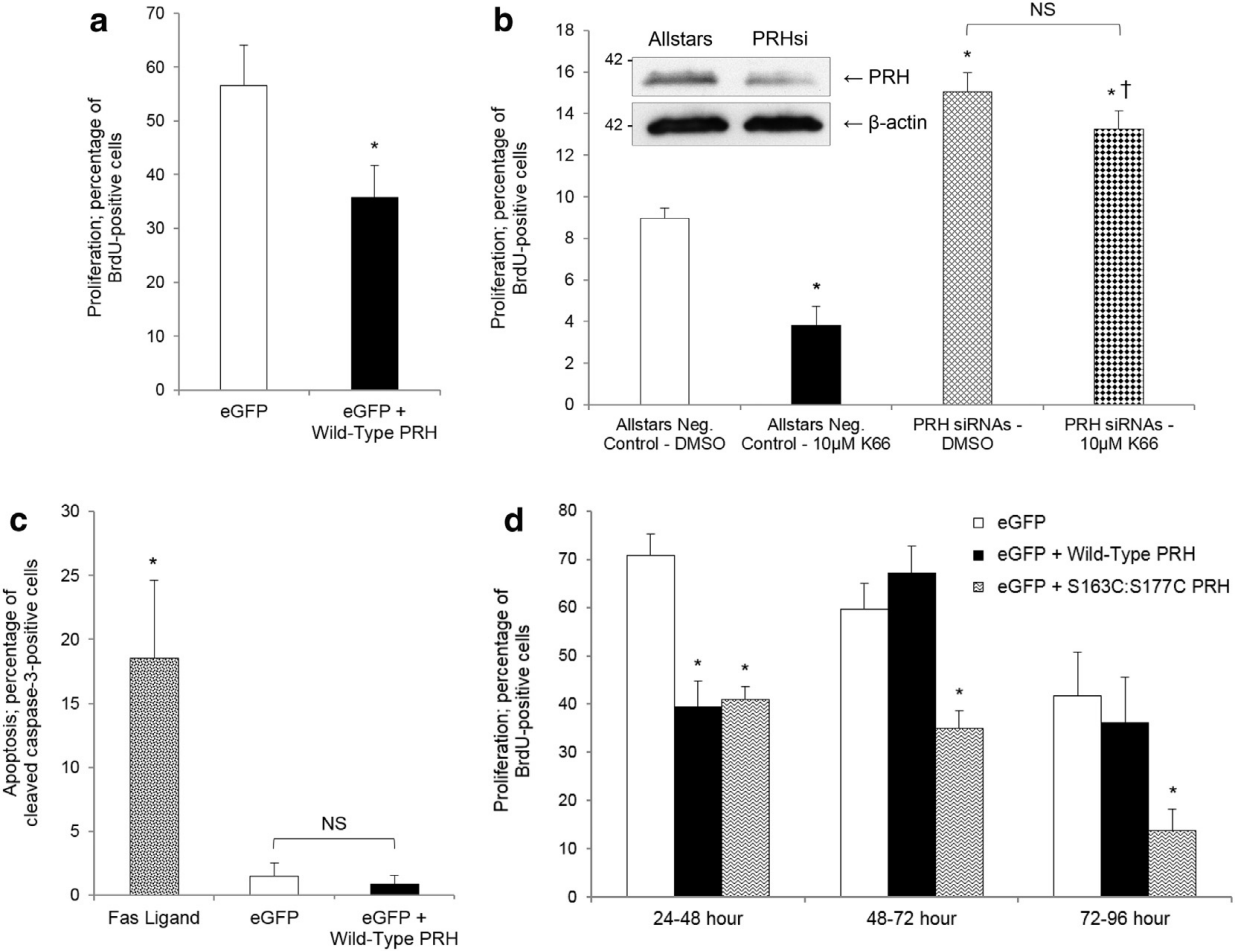
PRH inhibited VSMC proliferation. a, Quiescent rat aortic VSMCs were nucleofected with either eGFP, or eGFP and wild-type PRH expression vectors – co-transfection with eGFP enabled identification of cells expressing the transgene. Cells were stimulated with 10% FCS culture medium and proliferation assessed via quantification of BrdU incorporation between 24 and 48 h post-transfection. * indicates significant difference compared to eGFP, n = 7, p < 0.05, Students *t*-test. b, Serum-starved rat aortic VSMCs were subjected to nucleofection with either Allstars negative control or PRH siRNAs; cells were nucleofected twice, 72 h apart, to achieve significant depletion of PRH protein. Cells were stimulated with 20 ng/ml PDGF-BB and 20 ng/ml bFGF, and proliferation was assessed between 48 and 72 h post-transfection in the presence of either DMSO (vehicle control) or 10 μM K66. * indicates significant difference compared to Allstars Neg. Control - DMSO, † indicates significant difference compared to Allstars Neg. Control - 10 μM K66, n = 3, p < 0.05, ANOVA and Student Newman Keuls post hoc test. NS denotes not significant. Western blot shows validation of silencing of PRH, representative of n = 3. c, Quantification of apoptosis by immunofluorescence for cleaved caspase-3 48 h post-introduction of either eGFP, or eGFP and wild-type PRH expression vectors in rat aortic VSMCs. Fas ligand was used as a positive control. * indicates significant difference compared to eGFP and eGFP + wild-type PRH, n = 3, p < 0.05, ANOVA and Student Newman Keuls post hoc test. NS denotes not significant. d, Asynchronous rat aortic VSMCs over-expressing either wild-type PRH or S163C:S177C PRH were stimulated with 10% FCS culture medium, and proliferation quantified over a 96-h time course. * indicates significant difference compared to eGFP within the same time-frame, n = 3, p < 0.05, ANOVA and Student Newman Keuls post hoc test.

To determine the function of PRH in the context of VSMC proliferation, primary cultures of rat aortic VSMCs were subjected to ectopic overexpression or knockdown of PRH, then subsequent quantification of BrdU incorporation was performed. Introduction of PRH expression vectors via Amaxa nucleofection antagonised cell proliferation compared to eGFP-transfected cells (Fig. 2A). To achieve reduced (by 45 ± 8%, n = 3) expression of PRH double nucleofection of siRNA was required 72 h apart (Fig. 2B). The double nucleofection protocol using control Allstars siRNA reduced the rate of proliferation in response to PDGF-BB and bFGF to approximately 8% (Fig. 2B), this is lower than observed with single transfection (~ 20%, n = 3, Fig. 1A, B and G). SiRNA-induced silencing of PRH promoted cell replication (Fig. 2B). Together this demonstrated that PRH exerts an anti-proliferative action in cultured rat aortic VSMCs. Immunocytochemistry for cleaved caspase-3 demonstrated that PRH did not influence apoptotic cell turnover (Fig. 2C).

Previous studies have reported that mutation of Ser^163^ and Ser^177^ within the PRH homeodomain to cysteine residues is preventative of CK2-mediated phosphorylation, preserving DNA-binding potential, transcriptional regulation activity, nuclear retention and protein stability [19], [20]. To evaluate whether S163C:S177C PRH presents elevated or prolonged growth-inhibitory properties, cultured VSMCs were Amaxa nucleofected with wild-type or S163C:S177C PRH-encoding plasmids and proliferation monitored between 24 and 96 h post-nucleofection: up to 48 h, S163C:S177C PRH displayed a parallel anti-mitotic effect to wild-type PRH; though, unlike wild-type, exhibited the capacity to suppress cell division for up to 96 h (Fig. 2D).

### 3.3 Protein kinase CK2 promoted VSMC proliferation via a PRH-dependent mechanism in vitro

Primary cultures of rat aortic VSMCs were subjected to Amaxa nucleofection with PRH specific siRNAs, and subsequently treated with either 0.1% DMSO vehicle control or 10 μM K66 in 0.1% DMSO for 24 h. Treatment with K66 markedly attenuated the proliferative rate of cells transfected with negative control siRNAs, but its efficacy was lost in cells subjected to PRH silencing (Fig. 2B). These data highlight the salience of the CK2-PRH signalling axis in VSMC mitogenic signal transduction.

### 3.4 Protein kinase CK2 and PRH in cultured endothelial cells

**Fig. 3:**
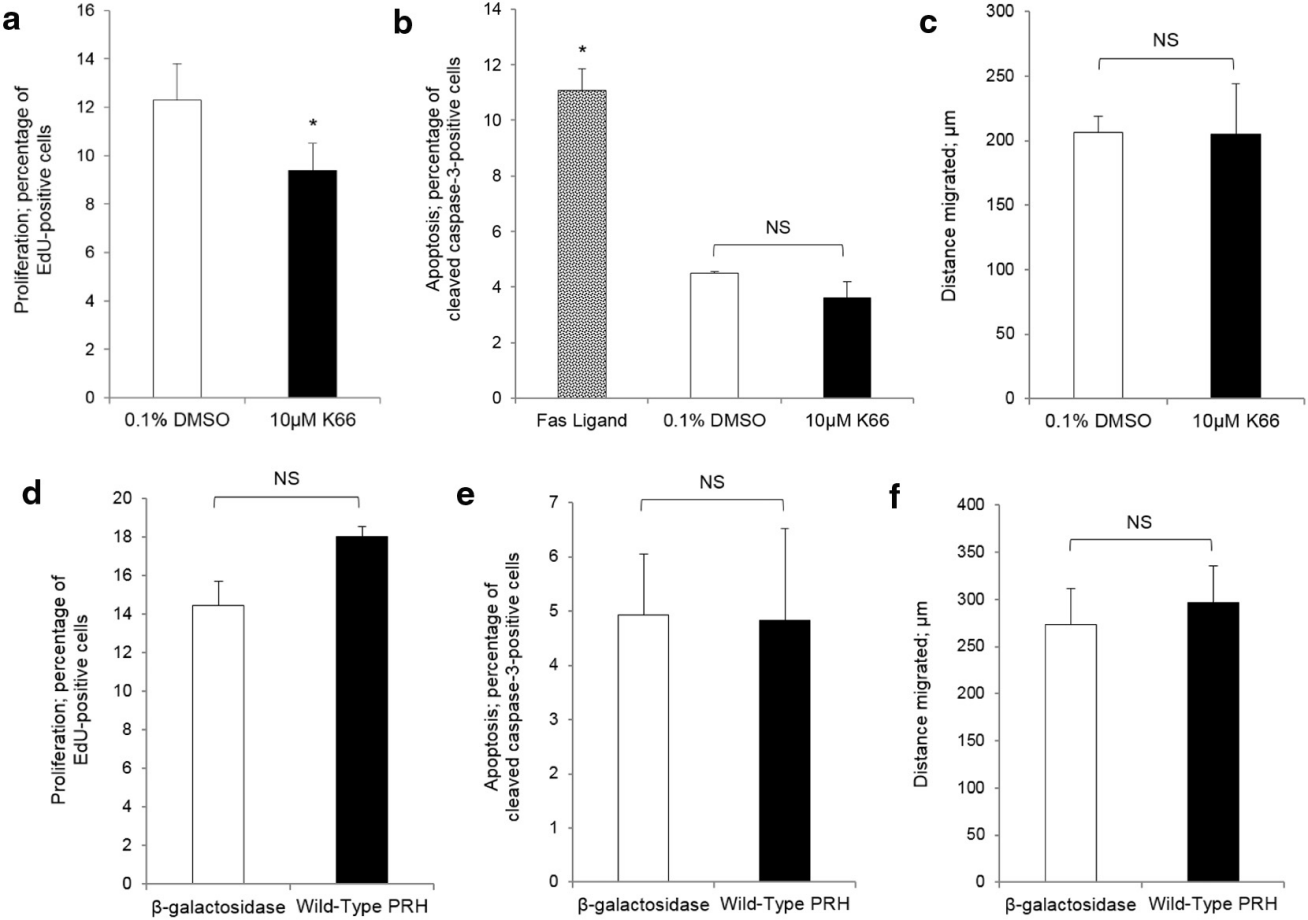
Effect of inhibition of protein kinase CK2 and overexpression of PRH on proliferation and apoptosis of HSaVECs. HSaVECs were cultured in endothelial cell growth medium. a, Quantification of proliferation by fluorescent labelling of incorporated EdU in HSaVECs treated with CK2 inhibitor (10 μM K66) or vehicle control (0.1% DMSO) for 24 h. * indicates significant difference compared to 0.1% DMSO, n = 3, p < 0.05, Students *t*-test. b, Quantification of apoptosis by immunocytochemistry for cleaved caspase-3 in HSaVECs treated with 10 μM K66 or 0.1% DMSO for 24 h. Fas ligand was used as a positive control. * indicates significant difference compared to 0.1% DMSO and 10 μM K66, n = 3, p < 0.05, ANOVA and Student Newman Keuls post hoc test. NS denotes not significant. c, Migration was quantified by scratch wound assay in HSaVECs treated with 10 μM K66 or 0.1% DMSO for 24 h. NS denotes not significant, n = 3. Quantification of proliferation by fluorescent labelling of incorporated EdU (d), apoptosis by immunocytochemistry for cleaved caspase-3 (e) or migration by scratch wound assay (f) in HSaVECs overexpressing PRH, n = 3. NS denotes not significant, Students *t*-test.

Thrombotic occlusion, intimal hyperplasia and atherosclerotic plaque development are direct consequences of endothelial dysfunction. In designing interventions to impair restenosis, one must consider whether the approach is detrimental to endothelial function or preventative of post-operative re-endothelialisation so as to not exacerbate disease progression. As inhibition of protein kinase CK2 and overexpression of PRH presented as viable antagonists of VSMC replication, and thus show potential for impairing neointima formation in a clinical setting, the risk of any adverse effects to the endothelium was evaluated. In both HUVECs (data not shown) and HSaVECs, treatment with 10 μM K66 marginally disrupted cell cycle progression yet did not influence apoptosis or migration as assessed by quantifying BrdU/EdU incorporation, immunofluorescence for cleaved caspase-3 and scratch wound assays, respectively (Fig. 3A–C). Introduction of PRH expression constructs via use of jetPEI-HUVEC transfection reagent or adenovirus-mediated delivery also did not significantly affect proliferation, apoptosis or migration (Fig. 3D–F).

### 3.5 CK2 inhibition impaired neointima formation ex vivo

**Fig. 4:**
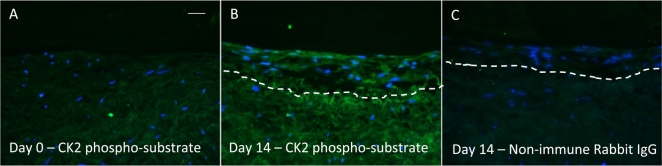
Enhanced CK2 activity as a result of neointima formation. Segments of human saphenous vein were subjected to organ for 0 or 14 days, n = 6, and subjected to immunofluorescence for phosphorylated CK2 substrates. a, vein segment at day 0 incubated with anti-phosphorylated CK2 substrate antibody, b, segment after 14 days of cultured incubated with anti-phosphorylated CK2 substrate antibody. c, segment after 14 days of cultured incubated with non-immune rabbit IgG (negative control). Phosphorylated CK2 protein (green) and nuclei are blue (DAPI). Scale bars represents 10 μm and apply to all panels. Dashed lines indicate intimal:medial boundaries. (For interpretation of the references to colour in this figure legend, the reader is referred to the web version of this article.)

**Fig. 5:**
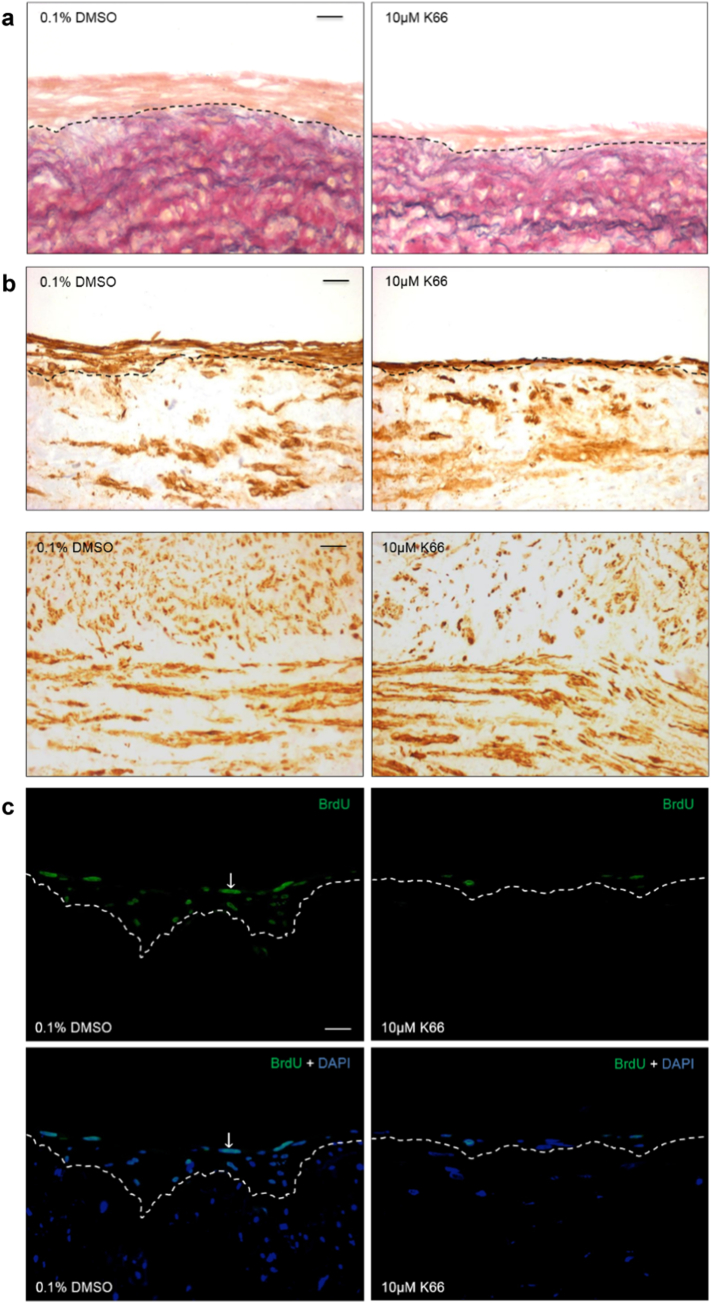
Treatment of human saphenous organ cultures with 10 μM K66 retarded neointima formation. Segments of human saphenous vein were subjected to organ culture in the presence of BrdU, and 10 μM K66 or vehicle control (0.1% DMSO) for 14 days, n = 6. a, EVG-stained sections of human saphenous vein organ cultures. b, Immunohistochemistry for intimal (upper panels) and medial (lower panels) α-smooth muscle cell actin (brown). c, Proliferation detected by immunofluorescence for incorporated BrdU (green). Nuclei are blue (DAPI) in lower panels. Arrows indicate positive cells. Scale bars represents 10 μm and apply to all panels. Dashed lines indicate intimal:medial boundaries. (For interpretation of the references to colour in this figure legend, the reader is referred to the web version of this article.)

**Fig. 6:**
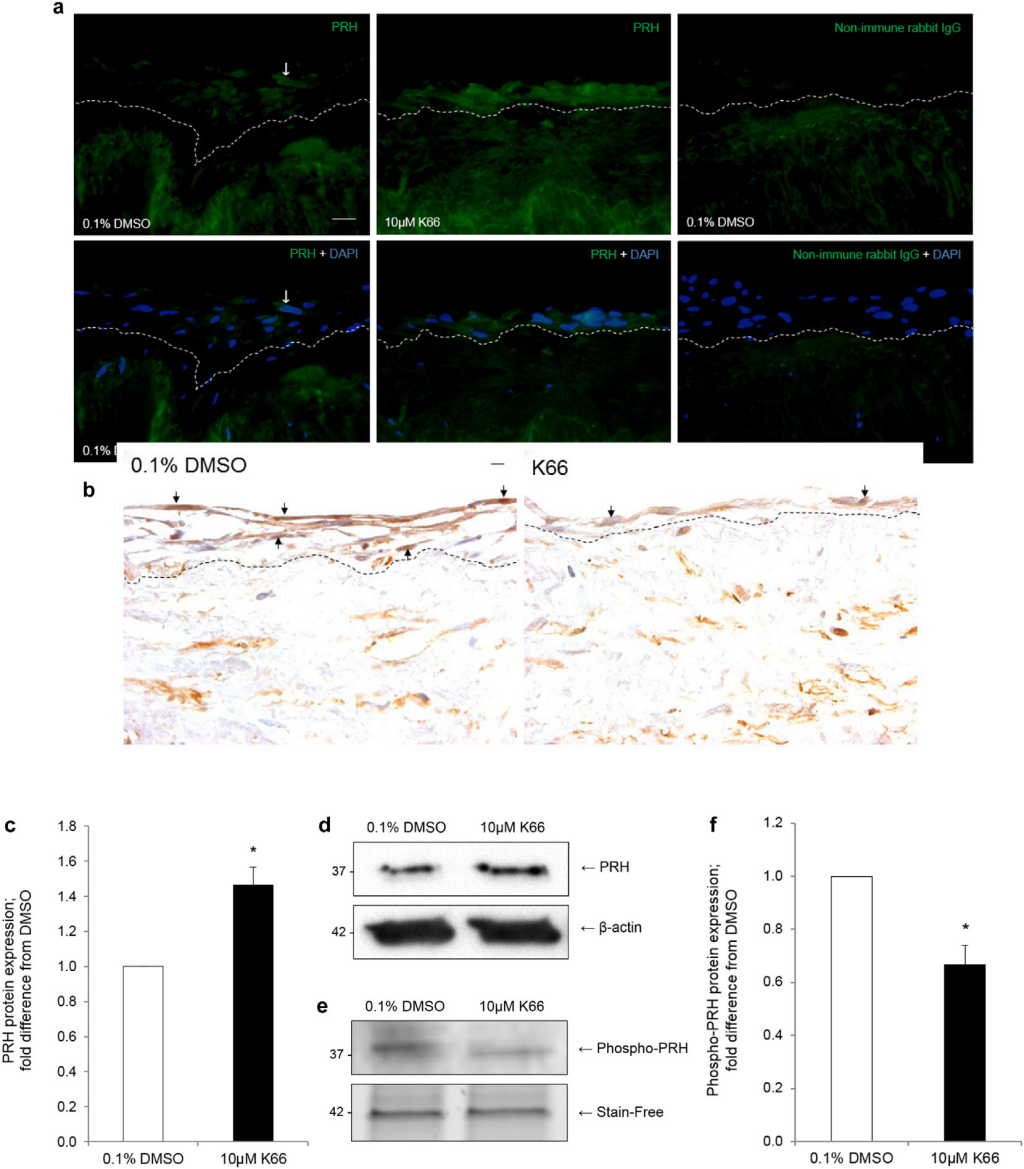
Accumulation of PRH and depletion of phospho-PRH in human saphenous vein organ cultures treated with 10 μM K66. Segments of human saphenous vein were subjected to organ culture in the presence of either 10 μM K66 or vehicle control (0.1% DMSO) for 14 days. a, Immunofluorescence for PRH protein (green). Nuclei are blue (DAPI) in lower panels. Arrows indicate positive cells. b, Immunohistochemistry for phospho-PRH protein (brown). Nuclei are-blue (haematoxylin). Scale bars represent 10 μm and apply to all panels. Dashed lines indicate intimal:medial boundaries. c, Quantification of PRH protein in whole tissue lysates by Western blotting; normalisation by β-actin. * indicates significant difference compared to 0.1% DMSO, n = 5, p < 0.05, one sample *t*-test. d, Representative Western blot for PRH protein in whole tissue lysates. e, Quiesced human saphenous vein VSMCs were stimulated with 20 ng/ml PDGF-BB and 20 ng/ml bFGF for 24 h in the presence of either 10 μM K66 or 0.1% DMSO. Representative Western blot for phospho-PRH protein. f, Quantification of phospho-PRH protein by Western blotting; normalisation by in-gel stain-free band. * indicates significant difference compared to 0.1% DMSO, n = 3, p < 0.05, one sample *t*-test. (For interpretation of the references to colour in this figure legend, the reader is referred to the web version of this article.)

Human saphenous vein organ cultures are a well-established model of neointima development which can be used for testing novel therapeutic interventions. Culture of saphenous vein for 14 days enhanced the amount of phosphorylated CK2 substrates, indicating enhanced CK2 activity (Fig. 4). Intact venous material was cultured for 14 days in the presence or absence of 0.1% DMSO vehicle control or 10 μM K66. Treatment with K66 dramatically reduced the average intimal thickness with respect to control (Fig. 5A and Table 1). No difference in neointimal cell or VSMC density was observed between control and treated samples (Fig. 4B and Table 1). K66 significantly decreased medial α-smooth muscle cell actin density (Fig. 5B and Table 1). Culture of human saphenous vein for 14 days causes an increase in α-smooth muscle cell actin density due to VSMC proliferation (day 14 vs. day 0, 11.5 ± 1.0 vs. 9.5 ± 1.0%, respectively). Interestingly, K66 suppressed this increase at day 14, and as a consequence the medial α-smooth muscle cell actin density was comparable to basal levels at day 0. Consequently, the decrease in α-smooth muscle cell actin density as a result of K66 result edin a similar density of VSMCs in the media as at day 0, and is unlikely therefore to lead to an adverse effect on vessel function. Quantification of BrdU incorporation demonstrated significant suppression of neointimal (Fig. 5C) and medial mitotic activity with K66 treatment though no response with regards to migratory activity (number of BrdU-negative cells per mm of neointima) was observed (Table 1). Immunofluorescence for cleaved caspase-3 revealed that the rate of apoptosis was extremely low and this was unaffected by the presence of K66. Both immunostaining of sections and Western blotting on whole tissues lysates identified an up-regulation in neointimal and total PRH protein expression in K66-treated material compared to control, respectively (Fig. 6A, C–D and Table 1). Histological staining further demonstrated a depletion of phospho-PRH protein in saphenous vein material treated with K66 suggesting effective inhibition of protein kinase CK2 (Fig. 6B and Table 1). In accordance with this, primary cultures of isolated human saphenous vein VSMCs were serum-starved for 72 h, then stimulated with 20 ng/ml recombinant human PDGF-BB and 20 ng/ml recombinant human bFGF for 24 h in the presence of either 10 μM K66 or 0.1% DMSO: Western blotting indicated a reduction in phospho-PRH protein in K66-treated samples (Fig. 6E–F).

**Table 1:** Human saphenous vein organ cultures treated with 0.1% DMSO vehicle control or 10 μM K66.

Parameter	0.1% DMSO	10 μM K66
Neointimal thickness/μm	39.5 ± 5.3	15.7 ± 3.5^a^
Cell density/cells/mm^2^	1211 ± 217	1524 ± 81
Neointimal αSMC actin density/%	48.9 ± 5.3	48.3 ± 5.1
Medial αSMC actin density/%	11.2 ± 1.5	9.8 ± 1.6^a^
Intimal proliferation/% BrdU-positive	64.9 ± 1.2	37.3 ± 1.6^a^
Medial proliferation/% BrdU-positive	6.3 ± 1.8	2.5 ± 1.3^a^
Migration/BrdU-negative cells/mm	16.8 ± 2.7	16.5 ± 1.6
Neointimal PRH protein/% PRH-positive	36.6 ± 6.1	60.4 ± 7.4^a^
Neointimal phospho-PRH protein/% phospho-PRH-positive	13.2 ± 2.2	9.1 ± 1.6^a^

a Statistically significant compared to 0.1% DMSO, n = 6, p < 0.05, Students *t*-test. SMC; smooth muscle cell.

### 3.6 Adenovirus-mediated delivery of PRH impaired neointima formation ex vivo

**Fig. 7:**
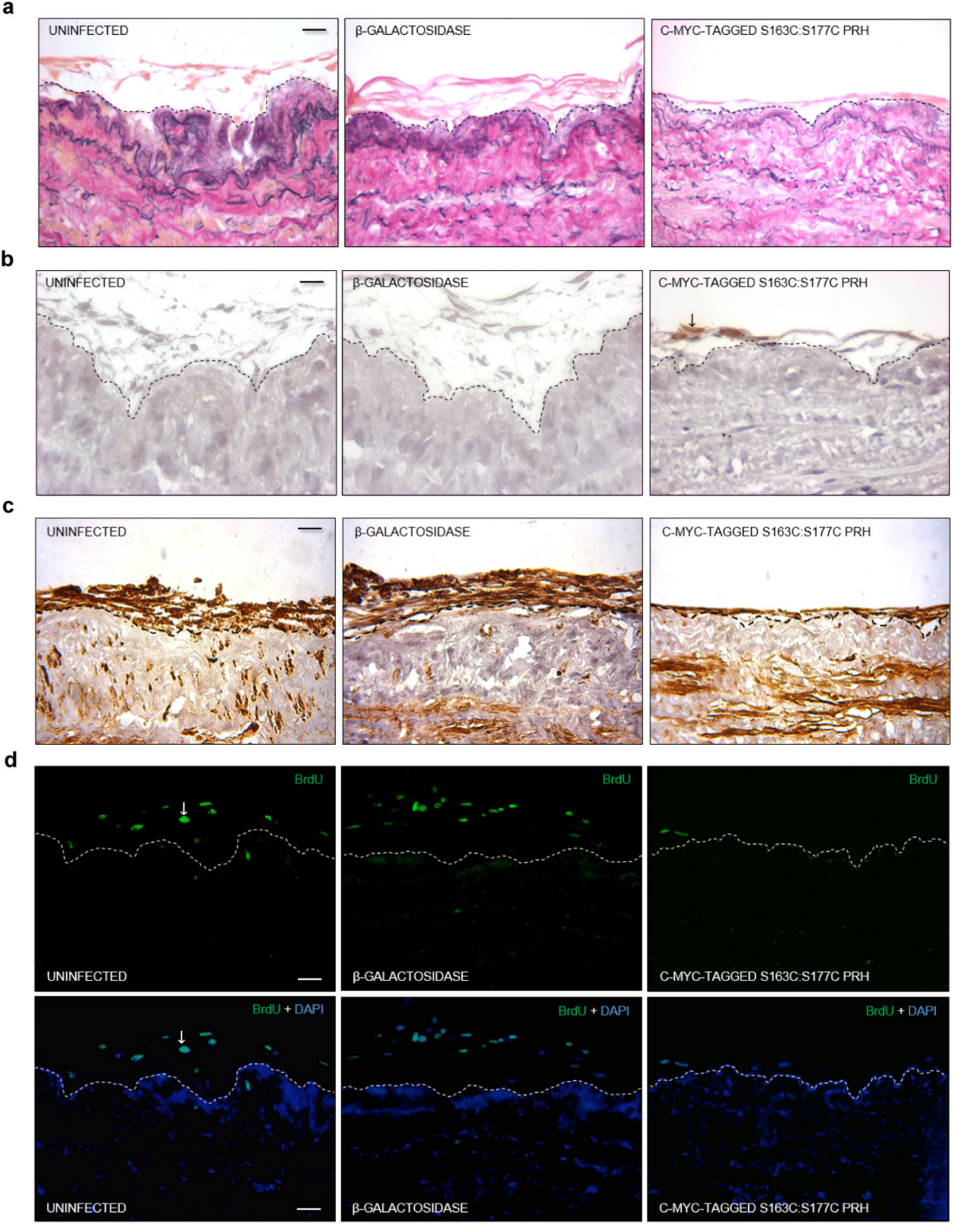
Adenovirus-mediated gene transfer of S163C:S177C PRH retarded neointima formation. Segments of human saphenous vein were subjected to organ culture in the presence of BrdU, with or without infection with Ad-β-galactosidase or Ad-c-myc-tagged-S163CS177C-PRH for 14 days, n = 5. a, EVG-stained sections of human saphenous vein organ cultures. b, Immunohistochemistry for c-myc-tagged protein (brown). c, Immunohistochemistry for α-smooth muscle cell actin (brown). d, Immunofluorescence for incorporated BrdU (green). Nuclei are blue (DAPI) in lower panels. Scale bars represents 10 μm and apply to all panels. Dashed lines indicate intimal:medial boundaries. Arrows indicate positive cells. (For interpretation of the references to colour in this figure legend, the reader is referred to the web version of this article.)

Intact segments of human saphenous vein were either uninfected or infected with β-galactosidase or c-myc-tagged S163C:S177C PRH-encoding vectors on days 0, 5, and 10 of a 14 day culture. Immunostaining for c-myc-tagged protein confirmed expression of c-myc-tagged S163C:S177C PRH at the luminal surface (Fig. 7B). Introduction of the S163C:S177C PRH transgene significantly reduced neointima formation with respect to uninfected control or venous material infected with β-galactosidase-encoding recombinant adenoviruses (Fig. 7A and Table 2). Neointimal cell or VSMC density was not affected by the introduction of the S163C:S177C PRH transgene (Fig. 7C and Table 2). Quantification of BrdU incorporation indicated a marked reduction in the percentage of proliferative, neointimal cells in vein segments infected with adenovirus expressing S163C:S177C PRH compared to controls (Fig. 7D and Table 2), though no influence on migration was observed (Table 2). Immunofluorescence for cleaved caspase-3 revealed no effect on apoptotic rate.

**Table 2:** Human saphenous vein organ cultures either uninfected or subjected to infection with adenoviruses encoding.

Parameter	Uninfected	β-galactosidase	S163C:S177C PRH
Neointimal thickness/μm	10.7 ± 1.2	12.5 ± 2.1	4.2 ± 0.9^a^
Cell density/cells/mm^2^	2308 ± 474	2319 ± 770	2765 ± 726
intimal αSMC actin density/%	48.4 ± 2.8	47.7 ± 3.2	46.9 ± 3.5
medial αSMC actin density/%	12.3 ± 1.4	11.3 ± 1.7	11.4 ± 1.5
intimal proliferation/% BrdU-positive	73.7 ± 5.0	70.2 ± 6.3	49.0 ± 5.7^a^
medial proliferation/% BrdU-positive	33.4 ± 9.5	32.6 ± 10.9	39.4 ± 11.9
migration/BrdU-negative cells/mm	5.4 ± 0.7	6.4 ± 0.9	6.1 ± 0.8

β-galactosidase or S163C:S177C PRH.

a Statistically significant compared to 0.1% DMSO, n = 5, p < 0.05, Students *t*-test. SMC; smooth muscle cell.

## 4 Discussion

Neointimal VSMC accumulation contributes considerably to vessel occlusion observed in both autologous vein graft degeneration [1], [2] and in-stent restenosis [26], [27]. In this study, the potential for retarding VSMC proliferation and hence ameliorating pathophysiological neointima formation via manipulation of the CK2-PRH signalling axis was evaluated. Utilising appropriate strategies for delivery of synthetic CK2 inhibitors may be of benefit to patients receiving vein grafts or drug-eluting stents.

Induction of VSMC proliferation with PDGF-BB and bFGF and culture of saphenous vein for 14 days led to enhanced CK2 activity (detected with anti-phosphorylated CK2 substrate antibody). Pharmacological inhibition of protein kinase CK2 with the synthetic compounds TBB and K66 significantly reduced proliferation in isolated VSMCs. The K66 compound was favoured for its putative low promiscuity and hence selected for the remainder of this study. The specificity of the K66 effect was supported by the observation that siRNA-induced silencing of the catalytic subunits of CK2 displayed comparable efficacy. Inhibition of CK2 activity via administration of K66 did not affect either VSMC apoptosis or migration. Importantly, K66 reduced levels of phosphorylated PRH protein and siRNA-mediated knockdown of PRH in K66-treated cultured VSMCs demonstrated that the anti-proliferative action of K66 required the presence of PRH. This suggests that phosphorylation and degradation of PRH are prerequisites for the growth-permissive effects of protein kinase CK2, as observed in human myeloid leukaemia K562 cells [19].

The multifunctional transcription factor PRH has been previously identified as a critical modulator of cell cycle progression, differentiation and development [14], but its role in regulation of VSMC proliferation and neointima formation was not fully elucidated. Sekiguchi et al. postulated that PRH may operate as a pathogenic factor by facilitating VSMC de-differentiation as its expression was induced with intimal thickening in conjunction with a molecular marker for de-differentiated, synthetic VSMCs [21]. In spite of this accumulation of PRH protein within the intima in vivo, our in vitro studies indicated that PRH did not facilitate cell cycle progression, but in fact revealed an anti-proliferative role for PRH. Most interestingly, the S163C:S177C PRH mutant displayed a prolonged anti-mitotic effect with respect to wild-type PRH. To clarify, mutation of the Ser^163^ and Ser^177^ residues within the PRH homeodomain is preventative of CK2-dependent phosphorylation, where phosphorylation abrogates DNA-binding potential and transcriptional regulation activity, reduces nuclear retention and decreases stability via proteolysis [19], [20]. Consequently, the S163C:S177C PRH mutant possesses enhanced stability [19], which is most likely the cause of the increased longevity of its anti-proliferative effect.

To ascertain whether interference with protein kinase CK2 activity or PRH expression would exacerbate endothelial dysfunction, HUVECs and HSaVECs were subjected to treatment with K66 or ectopic overexpression of PRH. K66 moderately retarded cell cycle progression without influencing cell survival and migration, whilst proliferation, migration and cell survival were unaffected by PRH gene transfer. This implies that K66 and PRH have very little effect on endothelial cell behaviour.

As demonstrated by Soyombo and colleagues [28], neointima formation is a phenomenon that can be successfully replicated in ex vivo human saphenous vein organ cultures, which is therefore a valuable technique for the testing of novel therapeutic interventions. Utilising this model, we demonstrated that K66 and adenovirus-mediated gene transfer of S163C:S177C PRH significantly retarded neointima formation. In both cases, this was exclusively attributed to their anti-proliferative activity as migration and cell viability were unaffected. Moreover, as expected, inhibition of protein kinase CK2 with K66 was associated with elevated PRH protein but decreased phosphorylated PRH levels in the saphenous vein organ cultures. This provides further support that CK2 regulates PRH protein levels and activity.

Regarding further translational aspirations, as protein kinase CK2 is ubiquitously expressed, systemic delivery of K66 would most likely result in undesirable off target side effects. Perivascular drug delivery systems may be employed for localised, sustained release of the K66 compound to a vascular graft. Sanders and colleagues [29], reported that delivery of anti-proliferative agents such as sunitinib – a multi-target receptor tyrosine kinase inhibitor – to porcine jugular veins could be achieved through application of a biocompatible hyaluronic acid-based hydrogel contained in a polyactide-*co*-glycolide perivascular wrap to impede drug diffusion into non-target extravascular tissue. Alternative drug delivery strategies include the use of drug-eluting nanoparticles or even drug-linked antibodies for direct administration of a compound to a target tissue [30]. The approaches discussed may all be considered for localised, sustained delivery of K66 to a grafted vasculature to prolong its patency.

To conclude, in this study, PRH has been identified as a novel inhibitor of VSMC proliferation; and its upstream regulator, protein kinase CK2, has additionally been documented to influence cell cycle progression. Inhibition of CK2 activity with the synthetic compound K66 and adenovirus-mediated ectopic overexpression of S163C:S177C PRH disrupted neointima development in human saphenous vein organ cultures. Progression into pre-clinical research may reveal the merit of such strategies in ameliorating saphenous vein graft stenosis and restenosis after stent implantation.
